# Pharmacological Inhibition of the Skeletal IKKβ Reduces Breast Cancer-Induced Osteolysis

**DOI:** 10.1007/s00223-018-0406-4

**Published:** 2018-02-17

**Authors:** Silvia Marino, Ryan T. Bishop, Patrick Mollat, Aymen I. Idris

**Affiliations:** 1 Department of Oncology and Metabolism, University of Sheffield, Medical School, Beech Hill Road, Sheffield, S10 2RX UK; 2 Bone and Cancer Group, Edinburgh Cancer Research Centre, MRC Institute of Genetics and Molecular Medicine, University of Edinburgh, Edinburgh, EH4 2XR UK; 3 Galapagos SASU, 102 Avenue Gaston Roussel, 93230 Romainville, France

**Keywords:** Osteolysis, IKKβ, Osteoclast, Breast cancer, Bone, Osteoclastogenesis

## Abstract

IKKβ has previously been implicated in breast cancer bone metastasis and bone remodelling. However, the contribution of IKKβ expressed by bone cells of the tumour microenvironment to breast cancer-induced osteolysis has yet to be investigated. Here, we studied the effects of the verified selective IKKβ inhibitors IKKβ^III^ or IKKβ^V^ on osteoclast formation and osteoblast differentiation in vitro and in vivo, human and mouse breast cancer cells’ support for osteoclast formation and signalling in vitro and osteolysis ex vivo and in immunocompetent mice after supracalvarial injection of human MDA-MB-231 conditioned medium or intra-cardiac injection of syngeneic 4T1 breast cancer cells. Pre-treatment with IKKβ^III^ or IKKβ^V^ prior to exposure to tumour-derived factors from human and mouse breast cancer cell lines protected against breast cancer-induced osteolysis in two independent immunocompetent mouse models of osteolysis and the ex vivo calvarial bone organ system. Detailed functional and mechanistic studies showed that direct inhibition of IKKβ kinase activity in osteoblasts and osteoclasts was associated with significant reduction of osteoclast formation, enhanced osteoclast apoptosis and reduced the ability of osteoblasts to support osteoclastogenesis in vitro. When combined with previous findings that suggest NFκB inhibition reduces breast cancer tumorigenesis and metastasis our present findings have an important clinical implication on raising the possibility that IKKβ inhibitors, as bone anabolics, osteoclast inhibitors as well as anti-metastatic agents, may have advantages over anti-osteoclasts agents in the treatment of both skeletal and non-skeletal complications associated with metastatic breast cancer.

## Introduction

NFκB is implicated in the development of breast cancer metastases [1–7] and its overexpression has been found to correlate with drug-resistance, resistance to radiotherapy and poor clinical outcome in breast cancer patients [8–11]. Many tumour- and bone-derived factors that have been implicated in the pathogenesis of breast cancer bone metastases including receptor activator of NFκB ligand (RANKL) and interleukin-1 (IL-1) activate NFκB [12–14]. More recently, there has been increasing interest in the therapeutic targeting of IKKβ, a key component of the canonical NFκB signalling pathway for the treatment of cancer-associated bone disease [14].

IKKβ plays a role in the regulation of bone remodelling [14–17]. Genetic inactivation or pharmacological inhibition of IKKβ inhibits osteoclastic bone resorption [1, 18–24] and promotes bone formation [25, 26]. IKKβ is also implicated in breast cancer bone metastasis [1] and we have recently reported that the indirect inhibition of IKKβ-mediated NFκB activation by the TAK1 (TAK1, TGF-beta-activated kinase 1) inhibitor Celastrol or the NFκB inhibitor Parthenolide reduced the development of osteolysis in a model of breast cancer bone metastasis [1, 20, 22, 27]. However, the effects of selective inhibition of IKKβ kinase activity on osteoblast and osteoclast changes associated with breast cancer bone metastasis have not been investigated.

Using the two verified selective IKKβ inhibitors IKKβ^III^ and IKKβ^V^ [28–30], we provide pharmacological evidence for the contribution of skeletal IKKβ to breast cancer-related bone cell activity and osteolysis. IKKβ^III^ and IKKβ^V^ protected against breast cancer-induced osteolysis in two immunocompetent mouse models of osteolysis and bone organ cultures derived from these mice. Further detailed functional and mechanistic studies showed that pre-exposure of osteoclasts and osteoblasts to these agents inhibited breast cancer cell-induced IKKβ activity and osteoclast formation and enhanced osteoblast differentiation. Based on these findings, we concluded that selective pharmacological inhibition of IKKβ signalling in the bone microenvironment might have a potential role in protecting the skeleton from the osteolysis associated with advanced breast cancer.

## Materials and Methods

### Materials

The selective IKKβ inhibitors IKKβ^III^ (401480) and IKKβ^V^ (401482) were purchased from Calbiochem (Dorset, UK) and dissolved in dimethyl sulfoxide (DMSO) for in vitro and ex vivo studies. All solvents and reagents were purchased from Sigma-Aldrich (Dorset, UK) unless otherwise stated. Tissue culture medium was obtained from Invitrogen (Paisley, UK). Human macrophage colony-stimulating factor (M-CSF, 416-ML-050) was purchased from R&D Systems (Abingdon, UK). RANKL was a gift from Patrick Mollat (Galapagos SASU, France) [16]. Foetal bovine serum (FBS) was obtained from GE Healthcare. Vitamin C (ascorbic acid) was obtained from BDH Laboratory Supplies (Dorset, UK). The following antibodies were used for Western blotting and immunostaining: anti-pIκB (ser32, 2859), anti-IκB (4812) from Cell Signalling Technology (Boston, MA, USA) and anti-β-actin (A5060) from Sigma-Aldrich (Dorset, UK).

### Cancer Cell Lines and Conditioned Medium

Human MDA-MB-231 (MDA-231) and mouse 4T1 breast cancer cells were purchased from ATCC (Manassas, VA) and cultured in standard Dulbecco’s modified essential medium (D-MEM) supplemented with 10% foetal calf serum (FCS), penicillin and streptomycin. For studies involving conditioned medium, cells were cultured in standard medium until 80% confluent. Cells were then cultured in serum-free medium and were allowed to grow for a further 16 h before conditioned medium was removed, filtered (0.45 µm) and used fresh (10–20% v/v).

### Animal Experimentation

All procedures involving mice and their care were approved by and performed in compliance with the guidelines of Institutional Animal Care and Use Committee of University of Edinburgh (Scotland, UK). C57BL/6J and BALB/c mice were obtained from Harlan (UK).

### Intra-tibial Injection of Tumour Cells

Seven 4-week-old female BALB/c mice received intra-tibial injection cancer cells (5 × 10^4^ cells/20 µl PBS) in the left leg [31]. Animals were euthanised 14 days (4T1) post injection, and bones were analysed by micro-computed tomography (microCT, Bruker 1172 scanner).

### Supracalvarial Injection of Human MDA-231 Conditioned Medium

The effects of human MDA-231 breast cancer-derived factors on osteolysis in vivo were studied by using the method described in [32]. Briefly, 3-week-old wild-type female C57Bl/6 mice were injected over the calvarial bones with 50 µl of human MDA-231 conditioned medium for 7 consecutive days. Mice were scarified 3 days after the last injection and the skull was fixed in 4% paraformaldehyde and bone density was assessed using micro-computed tomography (microCT; SkyScan 1172 scanner, SkyScan, Belgium) at a resolution of 5 µm. No mice injected with human MDA-231 conditioned medium in this study exhibited any obvious physical signs of illness or inflammatory response.

### The Calvarial Organ Culture

**Fig. 1:**
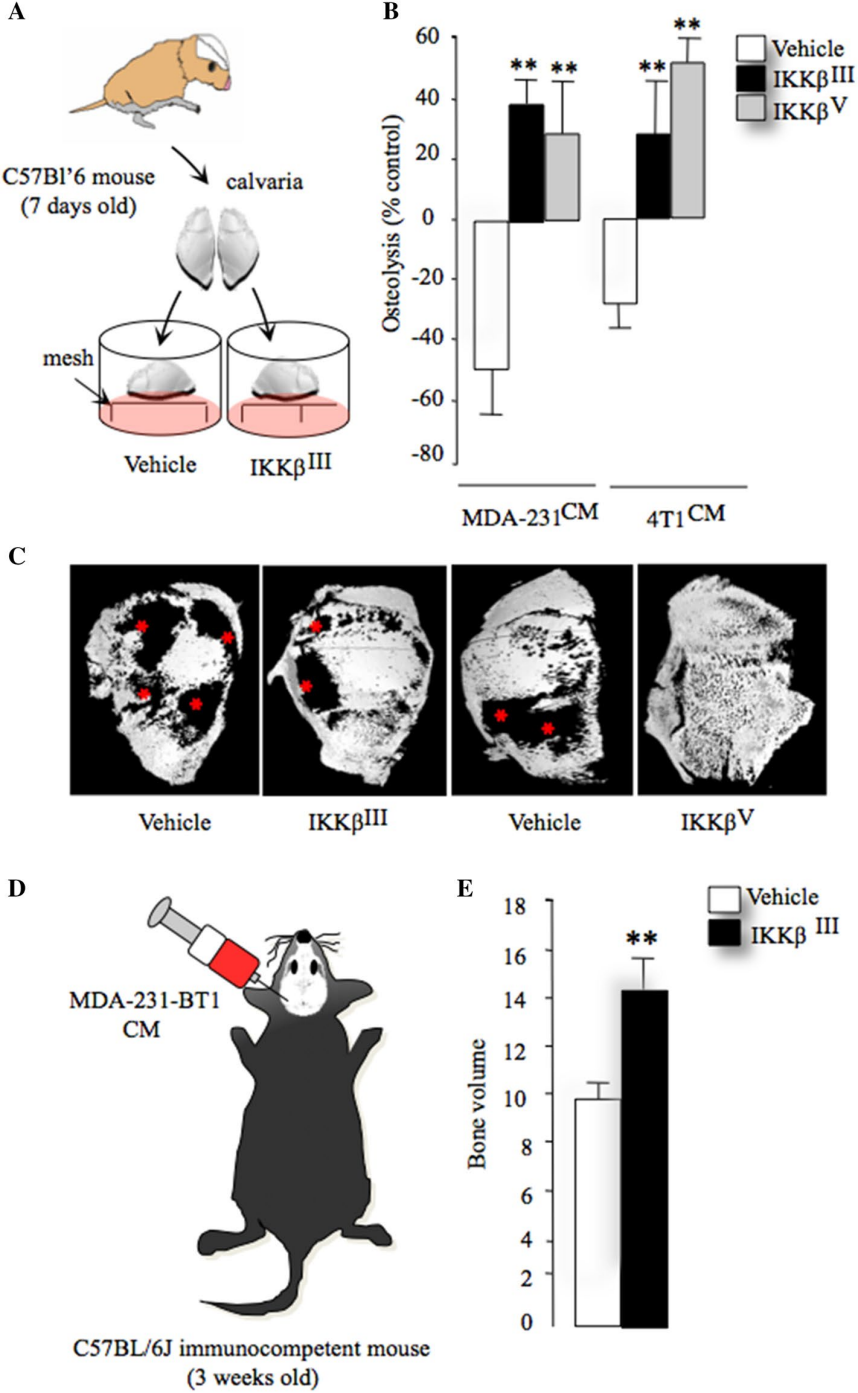
Inhibition of IKKβ reduces breast cancer—induced osteoclastogenesis and osteolysis. **a** Graphic representation of mouse calvaria organ co-culture system. **b** Total bone volume in mouse calvaria bone after exposure CM from human MDA-231 and mouse 4T1 breast cancer cells in the presence and absence of vehicle or the selective IKKβ inhibitors IKKβ^III^ or IKKβ^V^ (10 μM). **c** Representative photomicrographs of microCT scan of mouse calvaria bone from the experiment described in **a**, **b**. **d** Graphic representation of supracalvarial injection in adult immunocompetent mice. **e** Total bone volume (BV/TV, %) in mouse calvaria bone after exposure to CM from human MDA-231 breast cancer cells in the presence and absence of vehicle or the selective IKKβ inhibitor IKKβ^III^ (20 mg/kg/day) from the experiment described in panel **d** (*n* = 7). Data are mean ± SD, **p < 0.01 from vehicle

The effects of human MDA-231 breast cancer or their derived factors on osteolytic bone damage ex vivo were studied by using an adaptation of the mouse calvarial organ culture previously described in [33]. Neonatal mouse calvarias were isolated from 7-day-old C57Bl/6 mice, incubated in standard alpha-MEM for 24 h and divided into equal halves along the median sagittal suture. Each calvarial half was placed into culture on stainless steel rafts in 48-well plates (Fig. 1a). Tissue culture medium was changed every 48 h and fresh control medium containing test agents and conditioned medium form human MDA-231 cells (20% v/v) was added and the cultures were terminated after 7 days. At the end of the cultures calvarial bone density was assessed using micro-computed tomography (microCT; Bruker 1172 scanner, SkyScan, Belgium) at a resolution of 5 µm as described in [34].

### Bone Histomorphometry

Bones were fixed in buffered 4% formaldehyde for methyl methacrylate embedding without decalcification. Tartrate-resistant acid phosphatase (TRAcP) staining was used to evaluate osteoclast parameters and toluidine blue staining for osteoblasts. Histomorphometric measurements were carried out on 5-µm-thick sections, with an interactive image analysis system (IAS 2000, Delta Sistemi, Rome, Italy). Nomenclature, symbols and units of morphometric bone variables were used as previously described [35].

### Osteoblast Cultures

Primary osteoblasts were isolated from the calvarial bones of 2-day-old mice by sequential collagenase digestion as previously described [36]. Osteoblasts were maintained in alpha-MEM supplemented with 10% FCS and left to adhere overnight. For bone nodule assay, osteoblasts were seeded into 12-well plates at 10 × 10^5^ cells per well in alpha-MEM, supplemented with 10% FCS, penicillin and streptomycin, β-glycerol phosphate (10 µM) and l-ascorbic acid (50 µg/ml). The cells were cultured for up to 21 days with replacement of the culture medium in the presence or absence of test substances every 48 h. At the end of the culture period, osteoblast number, differentiation and bone nodule formation were determined by Alamar Blue assay [37], alkaline phosphatase (Alk Phos) assay and alizarin red (ALZ) staining [38].

### Osteoclast Cultures

Osteoclast formation, survival and activity were studied using RANKL- and M-CSF-generated mouse osteoclasts and bone marrow cell—osteoblast co-cultures as previously described in [39]. Briefly, bone marrow (BM) cells were flushed from the long bones of 3–4-month-old mice and were plated into Petri dishes and incubated for 48 h in standard alpha-MEM supplemented with mouse M-CSF (100 ng/ml). Erythrocytes were removed and the adherent cells were washed with phosphate-buffered saline (PBS) and re-suspended in culture medium and counted. For osteoclast generation, the resulting M-CSF-generated bone marrow macrophages (osteoclast precursor cells) were plated into tissue culture plates (96-well plates, 15 × 10^3^ cells/well; 12-well plates, 150 × 10^3^ cells/well) in standard alpha-MEM supplemented with M-CSF (25 ng/ml) and RANKL (100 ng/ml) or in the presence of calvarial osteoblasts [8 × 10^3^ cells/well in 150 µl of standard alpha-MEM containing 1,25-(OH)_2_-vitamin D_3_ (10 nM)]. For studies involving tumour-derived factors, conditioned medium from these cultures was prepared as described above then added to osteoclast cultures at a concentration of 10% (v/v) in standard alpha-MEM. The cultures were terminated by fixation in 4% paraformaldehyde and washed with PBS. TRAcP staining was used to identify multinucleated osteoclasts and TRAcP-positive cells with three or more nuclei were considered to be osteoclasts and manually counted on a Zeiss Axiovert light microscope using a 10× objective lens.

### TUNEL Assay

Briefly, adherent and non-adherent osteoclasts were collected, fixed with 4% paraformaldehyde and cytospun into glass slides. Apoptosis was identified on the basis of characteristic changes nuclear morphology using TUNEL staining as previously described [40]. Cells were counterstained with 4,6-diamidino-2-phenylindole (DAPI, 1 µg/ml) for 3 min prior to analysis by fluorescence microscopy. Cells were scored as apoptotic on the basis of nuclear morphology. An average of five microscopic fields per treatment group was analysed (×200 magnification).

### Caspase Assay

Caspase-3/7 activation was measured using Apo-ONE® Homogeneous Caspase-3/7 Assay (Promega, UK), according to the manufacturer’s instructions.

### Quantitative PCR

Quantitative PCR (qPCR) was used to measure mRNA expression. mRNA was isolated and quantified using a NanoDrop (Thermo Scientific), and complementary DNA (cDNA) was generated using Invitrogen SuperScript III Reverse Transcriptase kit according to manufacturer’s instructions. Primers for mouse RANKL (forward primer: 5′-TGAAGACACACTACCTGACTCCTG-3′, reverse primer 5′-CCACAATGTGTTGCAGTTCC-3′); mouse OPG (forward primer: 5′-ATGAACAAGTGGCTGTGCTG-3′, reverse primer 5′-CAGTTTCTGGGTCATAATGCAA-3′) and mouse GAPDH (forward primer: 5′-CCTGAATTTTAAGCTACACACAGC-3′, reverse primer 5′-CTGGCACTGCACAAGAAGAT-3′) were designed using the Ensembl genome browser and Roche website. Gene expression was expressed as copy number per microgram of total RNA, and GAPDH was used for cDNA normalisation.

### Western Blotting

Western blot analysis was used to detect protein expression and phosphorylation in cultured breast cancer and bone cells. Briefly, cells were seeded in 12-well plates and maintained in standard media until confluent. Prior to stimulation with test agents or vehicle, cells were incubated in serum-free alpha-MEM medium for 60 min (osteoclast cultures) or 16 h (osteoblast cultures). Test agents or vehicle were prepared in serum-free media and were then added for the desired period of time. The cells were then gently scraped in standard lysis buffer (0.1% (w/v) SDS, 0.5% (w/v) sodium deoxycholate, 1% Triton X-100, 1 µM EDTA, 2% (v/v) protease inhibitor cocktail, 10 µM of sodium fluoride and 2% (v/v) phosphatase inhibitor cocktail. The lysate was incubated on ice for 10 min and centrifuged at 4 °C for 5 min. Protein concentration was determined using BCA assay (Pierce, USA). Total protein (30–70 µg) was resolved by SDS-PAGE on 12% polyacrylamide SDS gels, transferred onto PVDF membranes (Bio-RAD, UK) and immunoblotted with appropriate antibodies according to manufacturer’s instructions. The immunocomplexes were visualised by an enhanced chemiluminescence detection kit (Pierce, USA) using horseradish peroxidase-conjugated secondary antibody (Jackson labs, UK), and then visualised using chemiluminescence (Amersham, UK) on a Syngene Gene Gnome imaging system.

## Statistical Analysis

Data are presented as mean ± standard deviation (SD). Statistical comparisons were performed using unpaired two-sided Student’s *t* test, or analysis of variance (ANOVA) followed by Dunnett’s post hoc test (SPSS, V11, IBM, USA). A *p* value of 0.05 or below was considered statistically significant.

## Results

### Bone-Autonomous Inhibition of IKKβ Suppresses Breast Cancer-Related Osteolysis

We investigated whether pharmacological inhibition of IKKβ in bone cells affects osteolysis induced by tumour-derived factors. Exposure of calvaria organ cultures derived from immunocompetent mice to conditioned medium from human MDA-231 or mouse 4T1 breast cancer cells (Fig. 1a) caused significant osteolytic bone damage when compared to control. These effects were completely abolished in the presence of the selective IKKβ inhibitors IKKβ^III^ or IKKβ^V^ (1 µM) (Fig. 1b, c, *p* < 0.01). Interestingly, pre-treatment of calvaria organ culture with the selective IKKβ inhibitors IKKβ^III^ or IKKβ^V^ caused a significant increase in bone volume when compared to vehicle control. Next, we took advantage of an in vivo supracalvarial injection mouse model to assess osteolysis in response to MDA-231 conditioned medium in adult immunocompetent mice (Fig. 1d). Administration of the selective IKKβ inhibitors IKKβ^III^ (20 mg/kg/day) into mice one day prior to injection of conditioned medium from human MDA-231 (Fig. 1d) caused a significant increase of bone volume when compared to control mice (Fig. 1e, *p* < 0.01).

## Inhibition of IKKβ Reduces Breast Cancer-Induced Osteoclastogenesis

**Fig. 2:**
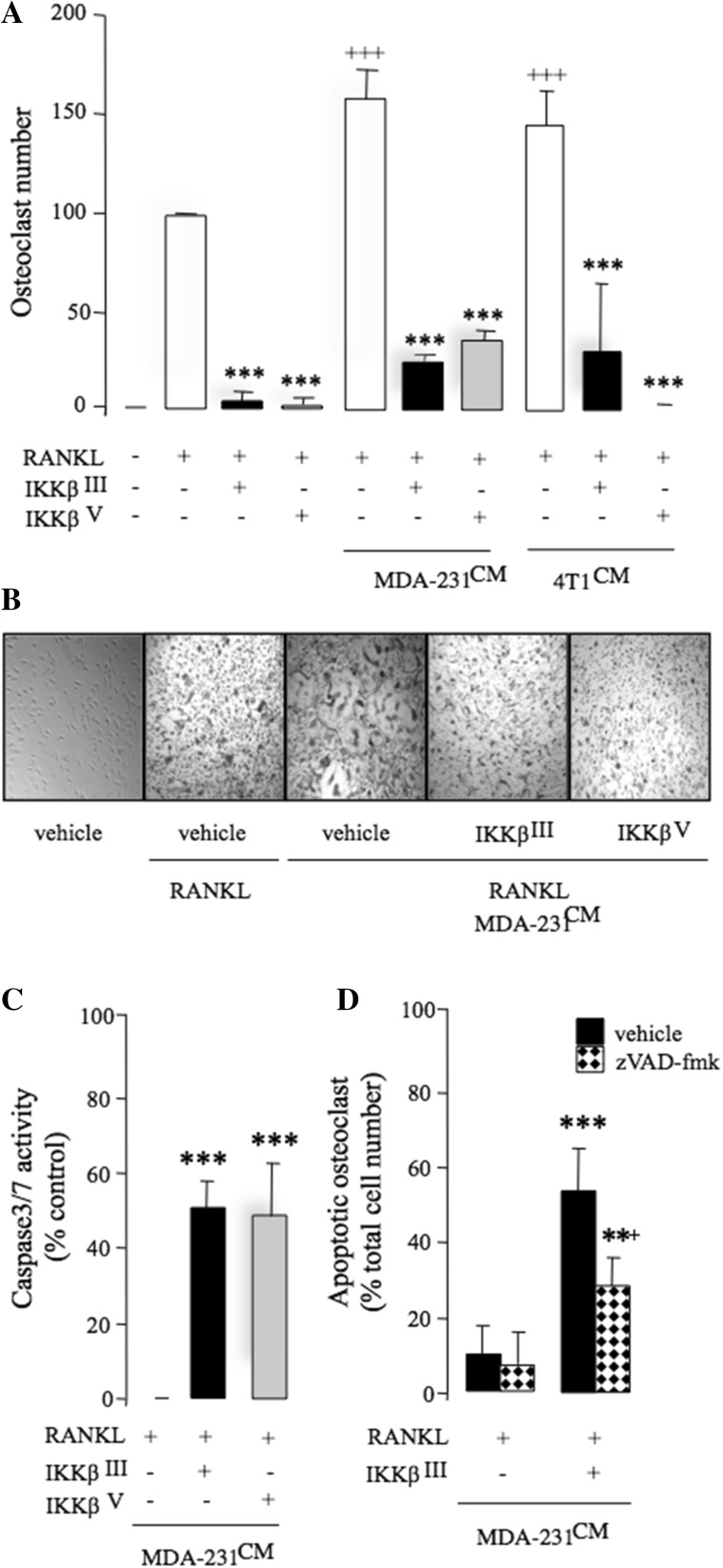
Inhibition of IKKβ prevents RANKL and breast cancer cell-induced osteoclastogenesis. **a** Number of osteoclasts in mouse M-CSF-dependent pre-osteoclast cultures after exposure to RANKL (100 ng/ml) and/or CM from human MDA-231 breast cancer cells (20% v/v) in the presence of vehicle (0.01% DMSO) or the selective IKKβ inhibitors IKKβ^III^ or IKKβ^V^ (3 μM). **b** Representative photomicrographs of M-CSF-dependent pre-osteoclasts and TRAcP-positive multinucleated osteoclasts from the experiment described in panel **a**. **c** In vitro osteoclast apoptosis as evidenced by caspase-3/7 activation in M-CSF- and RANKL-generated mature osteoclast cultures treated with IKKβ^III^ or IKKβ^V^ (10 μM) after 6 h. **d** In vitro osteoclast apoptosis as evidenced by DNA fragmentation in M-CSF- and RANKL-generated mature osteoclast cultures treated with IKKβ^III^ (10 μM) in the presence or absence of the caspase inhibitor zVAD-fmk (10 μM) after 24 h. Values are mean ± SD; **p < 0.05 and ***p < 0.001 from vehicle; ^+++^*p* < 0.001 from RANKL treated; ^+^*p* < 0.05 from IKKβ^III^ plus caspase inhibitor zVAD-fmk

Next, we tested the effects of pharmacological inhibition of IKKβ on breast cancer-induced osteoclastogenesis in vitro. As shown in Fig. 2a, exposure of mouse M-CSF generated osteoclast precursors to conditioned medium from breast cancer cells MDA-231 and 4T1 (20% v/v) enhanced RANKL-induced osteoclast formation (*p* < 0.001), and these effects were significantly reduced by the selective IKKβ inhibitors IKKβ^III^ or IKKβ^V^ (3 µM). Representative photomicrographs of osteoclasts from the experiment described are shown in Fig. 2b. None of these compounds affected the growth of M-CSF generated osteoclast precursors at the concentration tested (data not shown), thereby excluding the possibility that the inhibitory effect on osteoclast formation was mediated by a reduction in pre-osteoclast cell number. To investigate the effects of pharmacological inhibition of ΙΚΚβ on osteoclast survival in the presence of breast cancer-derived factors, we generated mature osteoclasts and exposed these cells to the selective IKKβ inhibitors IKKβ^III^ or IKKβ^V^ (10 µM) in the presence and absence of conditioned medium from human MDA-231 cells. IKKβ^III^ or IKKβ^V^ (10 µM) induced caspase-3/7 activation in mature osteoclasts within 6 h (Fig. 2c) and caused osteoclast apoptosis after 24 h (Fig. 2d), as evident by nuclear condensation and DNA fragmentation as measured by DAPI and TUNEL assays, respectively. Osteoclast apoptosis in these cultures was inhibited in the presence of the Caspase inhibitor zVAD-fmk (Fig. 2d), indicative of caspase involvement.

### Inhibition of IKKβ Reduces Breast Cancer-Associated Osteoblast Support for Osteoclastogenesis

**Fig. 3:**
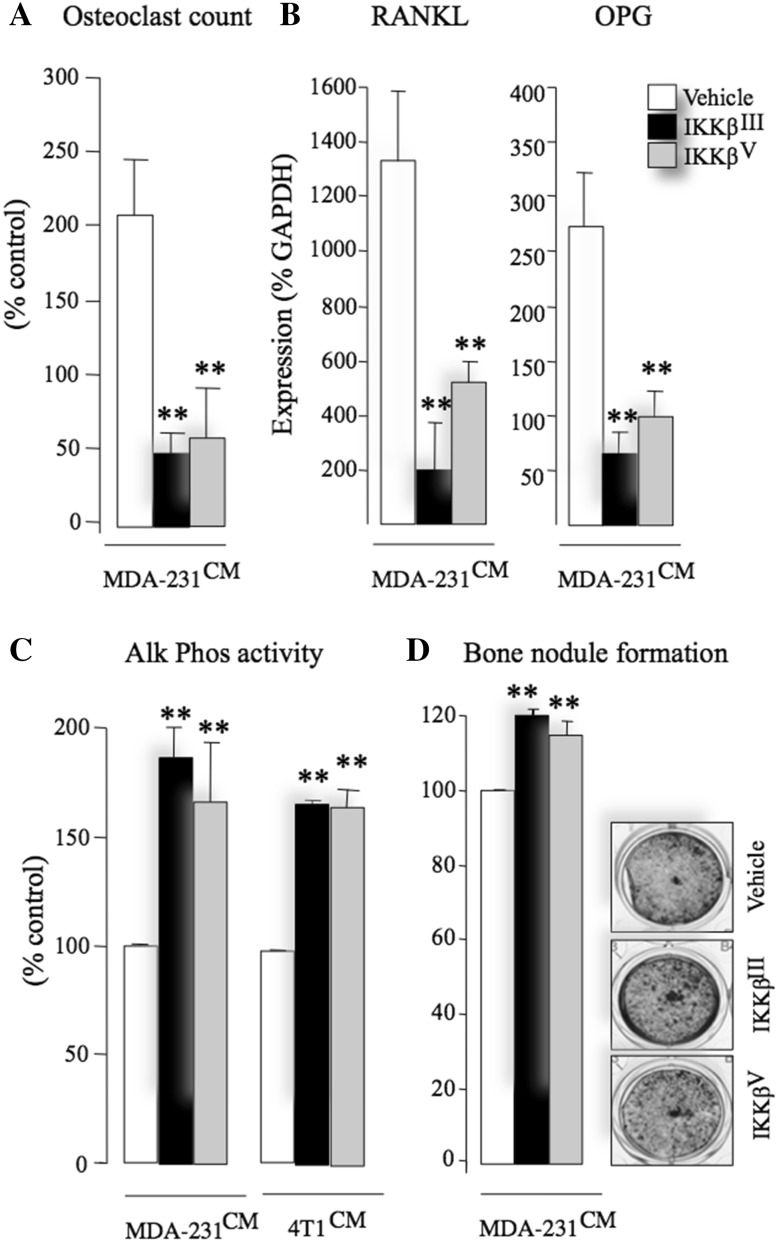
Inhibition of IKKβ enhances osteoblast maturation and reduces their support for osteoclastogenesis. **a** Number of osteoclasts in mouse osteoblasts—bone marrow cell cultures after exposure to CM from human MDA-231 breast cancer cells in the presence of vehicle (0.01% DMSO) or the selective IKKβ inhibitors IKKβ^III^ or IKKβ^V^ (3 μM). The average number of osteoclasts in vehicle-treated cultures in three independent experiments is 45 ± 6, 51 ± 12 and 72 ± 6 osteoclasts/well. **b** The mRNA expression of RANKL (left) and OPG (right) in mouse calvarial osteoblasts after exposure to CM from human MDA-231 breast cancer cells in the presence of vehicle (0.01% DMSO) or the selective IKKβ inhibitors IKKβ^III^ or IKKβ^V^ (10 μM). **c**, **d** Alkaline phosphatase activity (Alk Phos, **c**) and bone nodule formation (ALZ, **d**) in mouse calvarial osteoblasts after exposure to CM from human MDA-231 breast cancer cells in the presence of vehicle (0.01% DMSO) or the selective IKKβ inhibitors IKKβ^III^ or IKKβ^V^ (1 μM). Representative photomicrographs of osteoblast cultures from the experiment are shown in panel **d**, right. Data are mean ± SD, **p < 0.01 from vehicle

Osteoblasts enhance breast cancer-induced osteoclastogenesis by secreting various osteoclastic factors such as RANKL [41–43]. In this study, we tested the effects of IKKβ inhibition on osteoblast support for osteoclastogenesis in the presence of conditioned medium from the human MDA-231 breast cancer cells. Pre-treatment of calvarial osteoblasts with the selective IKKβ inhibitors IKKβ^III^ or IKKβ^V^ (3 µM) prior to the addition of mouse bone marrow cells and conditioned medium from the human MDA-231 (20% v/v) inhibited osteoclast formation from bone marrow-derived osteoclast precursors (Fig. 3a) and significantly reduced mRNA expression of RANKL (Fig. 3b, left) and OPG (Osteoprotegerin, Fig. 3b, right). Collectively, these results demonstrate that selective IKKβ inhibition in calvarial osteoblasts disrupts the ability of breast cancer cells to enhance osteoclastogenesis in the model described.

### Inhibition of IKKβ in Osteoblasts Increases Alkaline Phosphatase Activity

NFκB is implicated in the regulation of osteoblast differentiation and bone formation [25]. With this in mind, we examined the effects of the selective IKKβ inhibitors IKKβ^III^ or IKKβ^V^ on osteoblast proliferation and differentiation in the presence of conditioned medium from human MDA-231 and mouse breast cancer cells. Treatment of mouse calvarial osteoblasts with IKKβ^III^ or IKKβ^V^ (1.0 µM) increased alkaline phosphatase activity within 24 h (Fig. 3c), without affecting cell viability when compared to vehicle-treated control cultures (data not shown). Prolonged exposure to these agents enhanced bone nodule formation after 21 days (Fig. 3d) (*p* < 0.01). Representative photomicrographs of bone nodule formation from the experiment described are shown in Fig. 3d, right.

### Inhibition of IKKβ in Bone Cells Reduces Breast Cancer-Induced IκB Phosphorylation

**Fig. 4:**
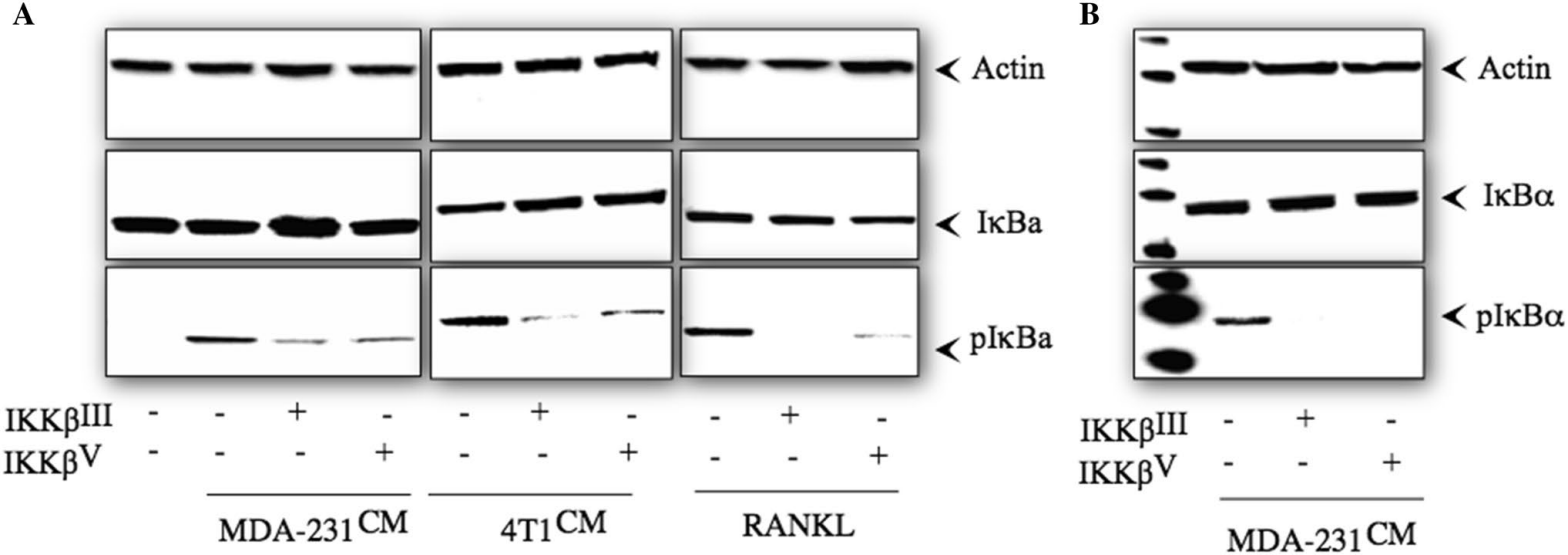
The selective IKKβ inhibitors IKKβIII or IKKβV reduce breast cancer-induced IκBα phosphorylation in osteoclasts and osteoblasts. **a** Western blot analysis of total and phosphorylated of IκB and actin in M-CSF dependent pre-osteoclasts after exposure to CM from human MDA-231 (left) and 4T1 (middle) breast cancer cells (20% v/v) and RANKL (100 ng/ml) in the presence and absence of the selective IKKβ inhibitors IKKβ^III^ or IKKβ^V^ (10 µM). **b** Western blot analysis of total and phosphorylated IκBα and actin in mouse calvarial osteoblasts after exposure to CM from human MDA-231 breast cancer cells (20% v/v) in the presence and absence of the selective IKKβ inhibitors IKKβ^III^ or IKKβ^V^ (10 µM)

Mechanistic studies in osteoclast precursors showed that exposure to the selective IKKβ inhibitors IKKβ^III^ or IKKβ^V^ (10 µM) for 1-h prior reduced IκB phosphorylation induced by MDA-231 or 4T1 conditioned medium after 15 min and RANKL (100 ng/ml) after 5 min (Fig. 4a). Similar inhibition of IκB phosphorylation was observed in calvarial osteoblasts exposed to MDA-231 conditioned medium for 15 min (Fig. 4b).

#### In Vivo Inhibition of IKKβ Reduces Breast Cancer-Induced Osteoclast and Enhances Osteoblast Number

**Fig. 5:**
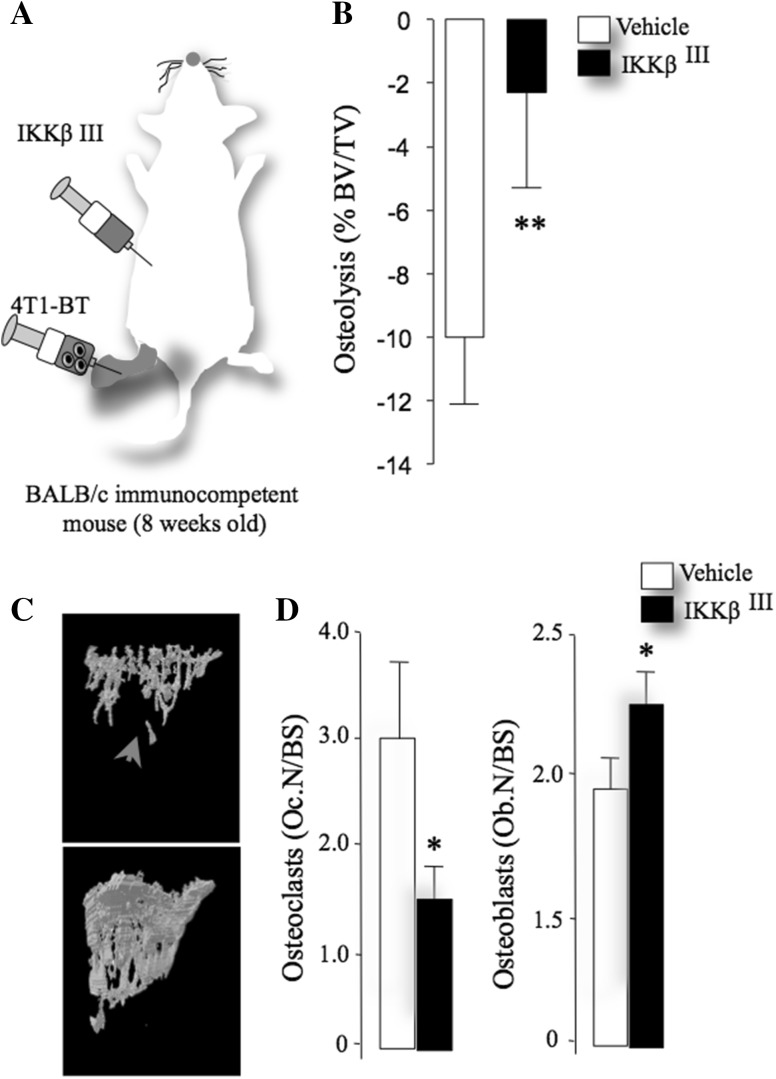
Selective IKKβ inhibition reduces breast cancer-induced osteolysis in vivo. **a** Graphic representation of intra-tibial injection of the mouse breast cancer cells 4T1 in immune-competent BALB/c mice pre-treated with vehicle (DMSO/PBS: 0.01:10) or IKKβ^III^ (20 mg/kg/day) for 12 days (*n* = 7). **b** Total bone volume (BV/TV) in tibial metaphysis of mice from the experiment described in panel **a**. **c** Representative photomicrographs of microCT scans of bone from the experiment described in panels **a**, **b**. Arrowhead denotes osteolysis. **d**, **e** In vivo number of osteoclasts (Oc.N/BS, left) and osteoblasts (Ob.N/BS, right) per bone surface (BS) from the experiment described in panels **a**–**c**. Values are mean ± SD; *p < 0.05 and **p < 0.01 from vehicle plus breast cancer cells

Using the intra-tibial 4T1 model of local osteolysis, we validated the effects of the selective IKKβ inhibitor IKKβ^III^ on osteoblast and osteoclast changes associated with breast cancer. IKKβ^III^ (20 mg/kg/day) was administered in mice 1 day prior to intra-tibial injection of the 4T1 (4 × 10^3^) and continued to day 10 (Fig. 5a). Detailed microCT and histomorphometric analysis of bone at the tibial metaphysis showed that IKKβ^III^ significantly inhibited osteolytic bone damage (Fig. 5b, c), reduced osteoclast number (Fig. 5d, left panel) and increased osteoblast number (Fig. 5d, right panel).

## Discussion

Bone is a common metastatic site for malignant breast cancer cells, and bone metastases are a cause of morbidity in advanced breast cancer patients [42, 44, 45]. Osteolytic bone metastases in advanced breast cancer patients result from breast cancer cells in the skeleton releasing factors that disrupt the function of osteoclasts and osteoblasts [42, 44, 45]. Various tumour-derived factors regulate breast cancer-induced osteolysis and bone cell activity by activating the canonical IKK/NFκB signalling pathway [1, 14, 21]. IKKβ also plays a key role in the bone remodelling and bone cell activity [1, 19, 21, 22, 24–26, 46] and we have recently shown that inhibition of the IKKβ/NFκB signalling pathway by using the TAK1 inhibitor Celastrol or the proteasome inhibitor Parthenolide reduced the development of osteolysis in a model of bone metastasis [1, 21, 22, 27].

Cells of the tumour microenvironment in the skeleton, particularly osteoclasts and osteoblasts, play a critical role in the regulation of breast cancer-induced osteolysis [42, 43]. However, previous studies that have implicated IKKβ in the regulation of osteolytic breast cancer metastasis were performed in immunodeficient mice treated with agents that indirectly inhibit the canonical IKKβ activity [1, 21, 22, 27]. To examine the contribution of IKKβ in osteoblasts and osteoclasts to breast cancer-osteolysis, we utilised the verified IKKβ inhibitors IKKβ^III^ or IKKβ^V^ [28–30] together with a number of in vivo, ex vivo and in vitro experiments using immunocompetent mice, organs and cells. An advantage of this approach is that the tumour microenvironment in the skeleton is not immunocompromised.

Our present results showed that pharmacological inhibition of IKKβ in host cells reduced breast cancer-induced osteolysis by disrupting breast cancer cell crosstalk with osteoblasts and osteoclasts. The evidence for osteoblast-specific IKKβ in these processes comes from the experiments that demonstrated that pre-exposure of osteoblasts to IKKβ inhibitors tested significantly reduced breast cancer-induced osteoclast formation in osteoblast–osteoclast co-cultures. Breast cancer cells secrete various factors that increase RANKL and inhibit OPG expression by osteoblasts. Mechanistic studies in osteoblasts confirmed the role of osteoblast-specific IKKβ in the reciprocal interaction between osteoblast and breast cancer cells by showing that pre-exposure of osteoblasts to IKKβ inhibitors reduced RANKL and OPG production by osteoblasts and these effects were accompanied with significant inhibition of IκB phosphorylation.

Breast cancer cells promote osteolysis by directly stimulating osteoclast formation [12, 13, 43, 47]. Studies in osteoclast cultures showed that selective pharmacological inhibition of IKKβ inhibited both breast cancer- and RANKL-induced osteoclast formation and caused osteoclast apoptosis in mature osteoclast cultures. This confirms that selective inhibition of IKKβ in mature osteoclasts and their precursors inhibits osteoclast formation and survival acting directly on osteoclasts and their precursors. Significant inhibition of osteoclast number was also observed in mice pre-treated with IKKβ^III^ prior to injection with 4T1 cells. It is important to note, however, that both IKKβ inhibitors tested prevented osteoclastic bone resorption in vitro but these effects were likely due to induction of apoptosis and cell death rather than a specific effect on osteoclast function (data not shown). Further mechanistic studies in mature osteoclasts confirmed the mechanism of action by which IKKβ^III^ and IKKβ^V^ exerted their anti-osteoclast effects by preventing RANKL-induced IκB phosphorylation and partial inhibition of these same pathways when activated with tumour-derived factors from the human MDA-231 breast cancer cells. Non-specific effects in M-CSF signalling were excluded by the fact that these agents did not inhibit M-CSF-induced signalling (data not shown) or cell viability in cultures of M-CSF-dependent osteoclast precursors (data not shown).

The treatment of breast cancer-associated bone disease is predominantly based on drugs that inhibit osteoclast formation and function and induce mature osteoclast apoptosis [42, 44, 45]. Whilst osteoclast inhibitors such as Bisphosphonates and Denosumab have been shown to be effective in the secondary prevention of skeletal-related events associated with various cancers including breast cancer [48–50], these agents have no significant impact on bone formation [48, 51].

Breast cancer cells in bone promote osteolysis by inhibiting osteoblast differentiation [12, 13, 43, 47] and previous studies reported that inhibition of IKK-mediated NFκB activity in osteoblasts increased osteoblast differentiation in vitro and promoted bone formation in vivo [19, 21, 22, 24–26]. Our current results expand and complement these observations by showing that selective inhibition of IKKβ kinase activity in osteoblasts was accompanied with marked increase in the ability of calvarial osteoblasts to differentiate and to form bone nodule in vitro. Together with the increase in osteoblast number that we observed in vivo, our in vitro data demonstrate that targeted inhibition of IKKβ in osteoblasts enhances their ability to mature even in the presence of breast cancer-derived factors. We, and others, have previously reported that inhibition of NFκB activity reduced bone loss through inhibition of osteoclast bone resorption and stimulation of osteoblast activity in inflammation- and ovariectomy-induced bone loss [19, 22, 24–26]. Our current data complement these studies and demonstrate that inhibition of IKKβ in the tumour microenvironment inhibited osteoclast activity, enhanced osteoblast differentiation and reduced bone loss associated with osteolytic breast cancer metastasis.

In conclusion, our present findings suggest that inhibition of the IKKβ/NFκB signalling in the bone microenvironment may have a potential role in protecting the skeleton from the osteolysis associated with breast cancer. In elucidating the mechanism of breast cancer-induced osteolysis, these findings together suggest that disrupting breast cancer cell—osteoblast–osteoclast communications through inhibition of IKKβ kinase activity in bone cells might protect against breast cancer-induced osteolysis by inhibiting excessive osteoclast formation and promoting osteoblast differentiation. When combined with previous studies, our pharmacological studies demonstrate that disruption of breast cancer- and RANKL-induced IKKβ activity in cells of the bone metastatic microenvironment resulted in pro-bone gain and anti-bone loss network of effects. These findings may have important clinical implications in raising the possibility that inhibitors of the canonical NFκB pathway, as osteoanabolics may have advantages over conventional anti-resorptive agents such as bisphosphonate [49, 52] and Denosumab [53, 54] for the treatment of osteolysis in advanced breast cancer patients given that they may encourage new bone regeneration. However, the potential use of NFκB inhibitors in cases of breast cancer with bone metastases needs to be carefully explored so that any treatment regime would take into consideration and exploit both, their cell-autonomous effects in the bone microenvironment and their direct effects on tumour. For that, further studies are needed and ongoing.
